# 14-3-3σ induces heat shock protein 70 expression in hepatocellular carcinoma

**DOI:** 10.1186/1471-2407-14-425

**Published:** 2014-06-12

**Authors:** Chia-Chia Liu, Yee-Jee Jan, Bor-Sheng Ko, Yao-Ming Wu, Shu-Man Liang, Shyh-Chang Chen, Yen-Ming Lee, Tzu-An Liu, Tzu-Ching Chang, John Wang, Song-Kun Shyue, Li-Ying Sung, Jun-Yang Liou

**Affiliations:** 1 Institute of Biotechnology, National Taiwan University, Taipei 106, Taiwan; 2 Institute of Cellular and System Medicine, National Health Research Institutes, Zhunan 350, Taiwan; 3 Department of Pathology and Laboratory Medicine, Taichung Veterans General Hospital, Taichung 407, Taiwan; 4 Department of Internal Medicine, National Taiwan University Hospital, Taipei 100, Taiwan; 5 Department of Surgery, National Taiwan University Hospital, Taipei 100, Taiwan; 6 Institute of Biomedical Sciences, Academia Sinica, Taipei 115, Taiwan; 7 Graduate Institute of Clinical Medical Science, China Medical University, Taichung 404, Taiwan; 8 Metabolomic Medicine Research Center, China Medical University, Taichung 404, Taiwan; 9 Graduate Institute of Basic Medical Science, China Medical University, Taichung 404, Taiwan

**Keywords:** 14-3-3σ, β-catenin, Hepatocellular carcinoma, HSF-1, HSP70

## Abstract

**Background:**

14-3-3σ is implicated in promoting tumor development of various malignancies. However, the clinical relevance of 14-3-3σ in hepatocellular carcinoma (HCC) tumor progression and modulation and pathway elucidation remain unclear.

**Methods:**

We investigated 14-3-3σ expression in 109 HCC tissues by immunohistochemistry. Overexpression and knockdown experiments were performed by transfection with cDNA or siRNA. Protein expression and cell migration were determined by Western blot and Boyden chamber assay.

**Results:**

In this study, we found that 14-3-3σ is abundantly expressed in HCC tumors. Stable or transient overexpression of 14-3-3σ induces the expression of heat shock factor-1α (HSF-1α) and heat shock protein 70 (HSP70) in HCC cells. Moreover, expression of 14-3-3σ significantly correlates with HSF-1α/HSP70 in HCC tumors and both 14-3-3σ and HSP70 overexpression are associated with micro-vascular thrombi in HCC patients, suggesting that 14-3-3σ/HSP70 expression is potentially involved in cell migration/invasion. Results of an *in vitro* migration assay indicate that 14-3-3σ promotes cell migration and that 14-3-3σ-induced cell migration is impaired by siRNA knockdown of HSP70. Finally, 14-3-3σ-induced HSF-1α/HSP70 expression is abolished by the knockdown of β-catenin or activation of GSK-3β.

**Conclusions:**

Our findings indicate that 14-3-3σ participates in promoting HCC cell migration and tumor development via β-catenin/HSF-1α/HSP70 pathway regulation. Thus, 14-3-3σ alone or combined with HSP70 are potential prognostic biomarkers for HCC.

## Background

The 14-3-3 family of proteins regulates multiple cellular processes with a highly conserved homology among all eukaryotic cells [1,2]. The 14-3-3σ isoform (also known as stratifin, SFN) sequesters Cdc2-cyclin B and Cdc/Cdk complexes in the cytoplasm, thereby inducing a G2 cell cycle arrest [3,4]. Transcriptional expression of 14-3-3σ is directly activated by the p53 tumor suppressor protein [5]. Epigenetic silencing of the 14-3-3σ (*SFN*) gene via CpG methylation has been reported in various cancer cells, including: breast, lung, liver, gastric cancer, ovarian and prostate cancers [6,7]. However, an increasing number of studies have demonstrated that 14-3-3σ overexpression promotes tumor progression [8-15]. An earlier study has indicated that frequent hypermethylation of CpG islands and the elimination of 14-3-3σ expression is found in human hepatocellular carcinoma (HCC) [16]. In contrast, increased 14-3-3σ expression was found in a proteomic study conducted to identify HCC associated protein markers [17]. These results, suggesting a “bipolar” role for 14-3-3σ, may be due to specific tissues, cell types, signaling pathways or may depend on the tumor-associated microenvironment.

Several molecular markers, including heat shock protein 70 (HSP70), glypican 3 (GPC3) and glutamine synthetase (GS) were proposed as diagnostic markers of hepatocellular nodules in cirrhosis and early HCC [18-24]. Among these potential diagnostic factors, HSP70 is also considered as a drug target for cancer therapy [25,26]. HSP70 is an essential molecular chaperon and is activated in response to stress and cell survival protection. It is tightly controlled by the upstream transcriptional factor heat shock factor-1 (HSF-1). Recent studies have indicated that HSF-1 is a key determinant in cancer progression and increased HSF-1 expression promotes HCC tumor invasion and metastasis [27,28]. These results suggest that HSF-1 and HSP70 are involved in facilitating HCC tumor development. Although the molecular mechanism of HSF-1′s transcriptional regulation is not well elucidated, some evidence indicates that the activation of glycogen synthase kinase-3β (GSK-3β) signaling attenuates HSF-1 activity [29-31]. GSK-3β is a serine/threonine protein kinase and a dysregulation in GSK-3β signaling has been suggested to be critical in influencing HCC cell growth [32]. GSK-3β phosphorylates β-catenin and subsequently facilitates β-catenin ubiquitination/degradation [33]. Accumulation or mutations of β-catenin increase cell proliferation and are associated with tumor progression in HCC [34-37].

The aim of the present study is to evaluate the role of 14-3-3σ in HCC and to investigate the potential molecular targets of 14-3-3σ in modulating HCC development. Our data indicates that 14-3-3σ expression is elevated in HCC tumors and increased 14-3-3σ expression promotes HCC tumor formation. Moreover, we show for the first time that 14-3-3σ induces HSP70 expression which induces cell migration via a β-catenin/HSF-1 dependent pathway. Our results reveal that HSP70 is the downstream regulator of 14-3-3σ which modulates HCC development. A combination of 14-3-3σ with HSF-1 and/or HSP70 might therefore be considered as potential prognostic biomarkers for HCC.

## Methods

### Clinical specimens

Tissue samples were obtained from 109 HCC patients who had undergone surgery for tumor resection at Taichung Veterans General Hospital from January 1999 to December 2001. Twenty-nine patients (26.6%) developed tissue-proven metastases, 3 to 87 months after resecting the primary HCC. Slides from paraffin-embedded surgical specimens, including the metastatic tumors and primary tumors with surrounding non-cancerous liver parenchyma, were subjected to immunohistochemical (IHC) staining. The IHC staining results were compared with pathological features, clinical parameters, including Barcelona-Clinic Liver Cancer (BCLC) staging, and disease outcomes. This study was approved by the Institutional Review Board of Taichung Veterans General Hospital. The policy that no informed consents are required for using these de-linked samples for retrospective analysis was also approved by the Institutional Review Board.

### Immunohistochemical analysis

Procedure of IHC analysis was performed as previously described [38-41]. 14-3-3σ, HSF-1 and HSP70 expressions in paraffin-embedded tissues were detected by use of primary antibodies against 14-3-3σ (Bethyl Laboratories, Montgomery, TX), HSF-1 and HSP70 (Santa Cruz Biotechnology, Heidelberg, Germany). A negative control was prepared by using the same staining procedure without primary antibodies. The IHC staining intensity was semiquantitatively scored by the Quick-score (Q-score) method based on intensity and heterogeneity, as described previously [38-43]. Briefly, the Q-score of a given tissue sample is the sum of the intensity and heterogeneity scores and ranges from 0 to 7. A Q-score ≥2 was considered as overexpressed or positive expression and a Q-score <2 was considered normal or negative expression. Cases with <5% weakly stained specimens were considered as negative expression.

### Cell culture and stable cells

Huh-7 and SK-Hep1 cells were maintained in a humidified incubator with 5% CO_2_ at 37°C in Dulbecco’s modified Eagle’s medium (DMEM) (Gibco, Gaithersburg, MD) supplemented with 10% fetal bovine serum (FBS; Hyclone Thermo Fisher Scientific, Waltham, MA), 100 U/mL penicillin and 100 U/mL streptomycin. To establish stable cell lines, 14-3-3σ cDNA was amplified by PCR and subcloned into the p3XFlag-CMV vector. Huh-7 cells were transfected with p3XFlag-CMV (control) and 14-3-3σ tagged with Flag (14-3-3σ) by use of the Polyje™ transfection reagent according to the manufacturer’s instructions (SignaGen Laboratories, Rockville, MD). The transfected cells were selected using 500 μg/mL G418 (Biochrom AG, Berlin, Germany) for 4 weeks. Single colonies of control and 14-3-3σ stable clones (at least 4 in each cell line) were maintained in DMEM with 10% FBS and 200 μg/mL G418.

### Western blot analysis

Cells were lysed with ice cold RIPA buffer (0.5 mol/L Tris–HCl, pH 7.4, 1.5 mol/L NaCl, 2.5% deoxycholic acid, 10% NP-40, 10 mmol/L EDTA; Millipore, Temecula, CA) containing a protease inhibitor cocktail (Roche, Indianapolis, IN). Cell lysates were centrifuged at 16,100 *g* at 4°C for 20 minutes. Protein concentrations were determined and 20 μg of total proteins were applied to the gradient SDS-PAGE gel and immunoblotted onto PVDF membranes. The membranes were blocked, incubated with primary antibodies against Flag (Sigma-Aldrich, St. Louis, MO), 14-3-3σ (Abcam PLC, Cambridge, UK), HSF-1, β-catenin (Cell Signaling Technology, Beverly, MA) or HSP70 (Santa Cruz Biotechnology, Heidelberg, Germany), followed by an incubation with a secondary antibody conjugated horseradish-peroxidase in PBST. Protein levels were determined by the use of enhanced chemiluminescence reagents.

### Cell proliferation assay

Cell proliferation was analyzed using a 3-(4,5-dimethylthiazol-2-yl)-2,5-dipheny ltetrazoliumbromide (MTT) assay as previously reported [38]. Briefly, cells were seeded in 96-well plates at a density of 1500 cells/well for 0, 24, 48 and 72 hours. 20 μL of MTT (5 mg/ml) (Sigma, St. Louis, MO) was added to each well and incubated at 37°C for 3 hours. Subsequently, the MTT solution was removed, DMSO was added and the absorbance value (OD) of each well was measured at 570 nm with a reference wavelength of 690 nm.

### Quantitative real-time PCR

Total RNA was extracted by use of the RNAspin Mini Kit (QIAGEN, Alameda, CA) and cDNA was synthesized from 2 to 5 μg RNA by use of the random primers and SuperScript™ III Reverse Transcriptase cDNA Synthesis Kit (Invitrogen™ Life Technology, Carlsbad, CA). Quantitative real-time PCR using SYBR Green (Kapabiosystem, Woburn, MA) with specific oligonucleotide primers of HSF-1 and HSP0 (Additional file 1: Table S1) were detected by the AB 7900HT system (Applied Biosystems, Carlsbad, CA). Applied Biosystems Relative Quantification Manager Software version 1.2 was used to analyze the relative gene expression in each sample by the comparative cycle threshold (Ct) method. Gene expression was normalized to that of glyceraldehyde-3-phosphate dehydrogenase.

### Transfection and siRNA knockdown

Targeted knockdown of the 14-3-3σ gene expressions with siRNA was purchased from Invitrogen™ including scramble siRNA (Cat.No.12935-112). siRNA for targeting HSP70 (sc-29352) and HSF-1 (sc-35611) were purchased from Santa Cruz (Additional file 1: Table S2). Cell transfections with siRNAs were performed using Lipofectamine™ RNAiMAX (Invitrogen, Grand Island, NY) and harvested at the indicated time for further analysis.

### Migration assay

The cell migration assay was performed in a Boyden chamber with Bio-coat cell migration chambers (Becton Dickinson, Pont-de-Claix, France) as described previously [38]. Cells were trypsinized, re-suspended with 0.1% bovine serum albumin (BSA)–DMEM and added to the upper wells (5 × 10^4^ cells for Huh-7 stable cells, 2 × 10^4^ cells for SK-Hep1 cells). The cells then migrated to the bottom wells that contained medium with 100 μg/mL fibronectin (Becton Dickinson, Pont-de-Claix, France), epidermal growth factor (20 ng/mL), and 10% BSA. The cells remaining on the upper well were removed and the migrating cells in the bottom well were stained and fixed with 0.1% crystal violet containing 20% ethanol and 1% formaldehyde for 20 minutes. Efficiency of cell migration was quantified by counting the total number of migrating cells.

### Statistical analysis

One-way analysis of variance (ANOVA) was used to analyze differences among clinicopathological variables by 14-3-3σ, HSP70 and HSF-1 expression. A Student’s *t*-test was used to analyze differences between 2 experimental groups. A *P* value <0.05 was considered statistically significant and a *P* value of between 0.05 and 0.10 was considered marginally significant.

## Results

### Increased expression of 14-3-3σ in HCC tumors

**Figure 1:**
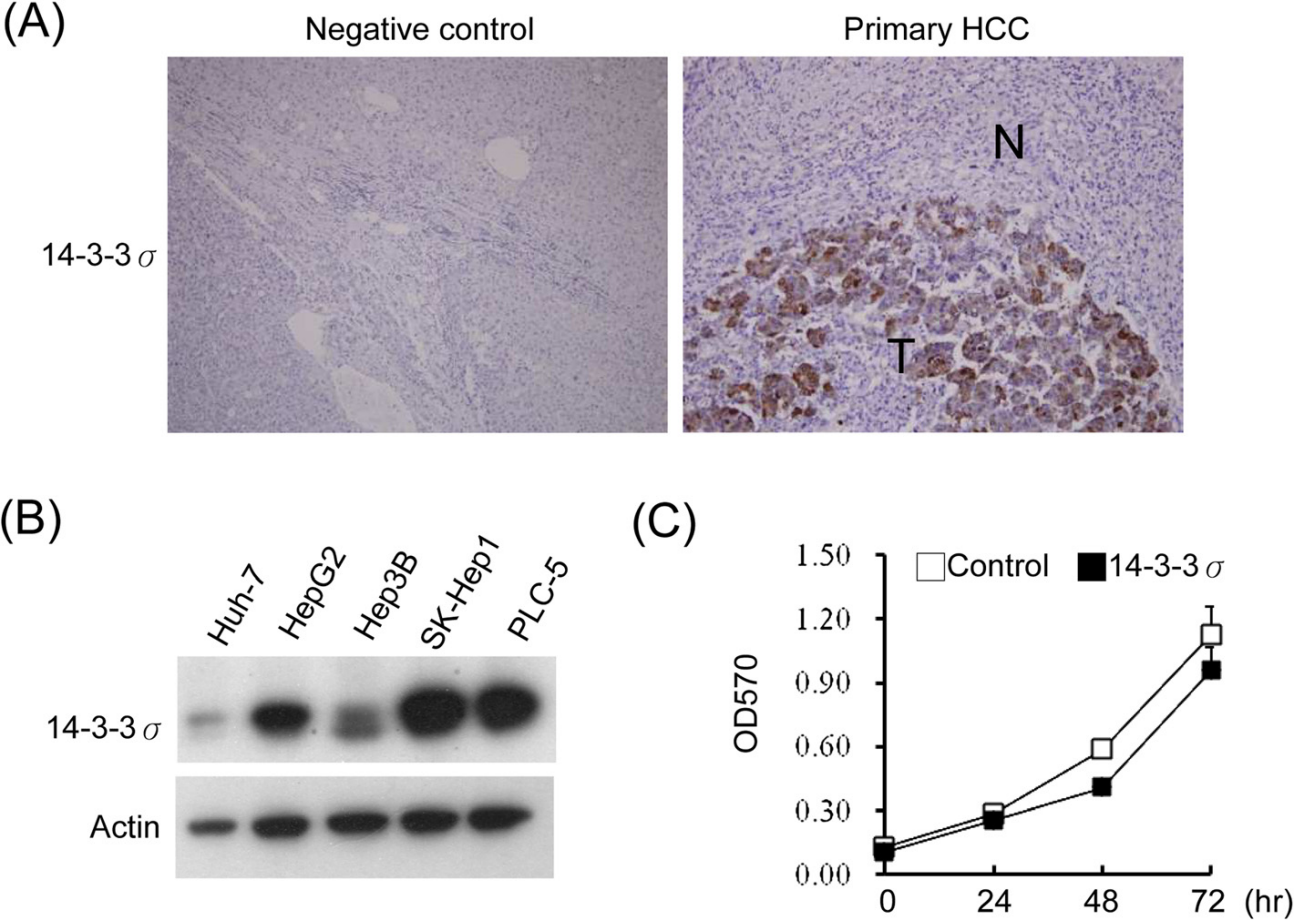
**14-3-3σ overexpression induces tumor growth. (A)** A represtative expression of 14-3-3σ in a primary HCC tumor and in a negative control examined by immunohistochemical analysis. T: tumor; N: non-cancerous cells. Original magnification: ×200. **(B)** 14-3-3σ expression levels in HCC cell lines were determined by Western blot analysis. Actin was used as a loading control. **(C)** The rate of cell proliferation was determined by an MTT assay. Scale bars: mean ± SD.

To determine the expression of 14-3-3σ in HCC, paraffin-embedded primary HCC tumors with surrounding non-cancerous tissues of 109 patients were examined by immunohistochemical staining. 14-3-3σ was undetected or was stained with the background in non-cancerous cells but had significantly increased expression in 84 of 109 (77.1%) primary HCC tumors (Figure 1A, right panel and Table 1). We next compared 14-3-3σ expression with the clinicopathological characteristics and found that increased 14-3-3σ expression was significantly associated with surgical margin (*P* = 0.008), capsular formation (*P* = 0.028) and micro-vascular thrombi (*P* = 0.001). These results indicate that expression of 14-3-3σ is associated with a more aggressive tumor behavior and a poor prognosis (Table 1).

**Table 1:** Correlation of 14-3-3σ and HSP70 with clinicopathological characteristics in primary HCC patients

**Parameters**	**14-3-3σ expression (Q-score ≥ 2) % (n)**	***p*-value**	**Hsp70 expression (Q-score ≥ 2) % (n)**	***p*-value**
Overall (n = 109)	77.1% (84)		60.6% (66)	
Age		0.080^†^		NS
< 60 years (n = 56)	83.9% (47)		66.1% (37)	
≥ 60 years (n = 53)	69.8% (37)		54.7% (29)	
Gender		NS		0.097^†^
Male (n = 82)	79.3% (65)		56.1% (46)	
Female (n = 27)	70.4% (19)		74.1% (20)	
Histology grade		NS		0.060^†^
1 (n = 7)	71.4% (5)		28.6% (2)	
2 (n = 77)	74.0% (57)		58.4% (45)	
3 (n = 25)	88.0% (22)		76.0% (19)	
Types of surgery		NS		NS
Wedge resection (n = 39)	69.2% (27)		56.4% (22)	
Segmentectomy (n = 54)	79.6% (43)		64.8% (35)	
Lobectomy (n = 16)	87.5% (14)		56.3% (9)	
Surgical margin		0.008*		NS
Free (n = 83)	71.1% (59)		61.4% (51)	
Involved (n = 26)	96.2% (25)		57.7% (15)	
BCLC staging		NS		0.079^†^
Not available (n = 5)				
Early (stage A1 to A4) (n = 56)	69.6% (39)		51.8% (29)	
Intermediate (stage B) (n = 46)	82.6% (38)		67.4% (31)	
Advanced (stage C) (n = 2)	100.0% (2)		100.0% (2)	
Tumor size		0.062^†^		0.062^†^
≥ 5.0 cm (n = 34)	88.2% (30)		73.5% (25)	
< 5.0 cm (n = 75)	72.0% (54)		54.7% (41)	
Tumor multiplicity		NS		NS
Single (n = 84)	78.6% (66)		63.1% (53)	
Multiple (n = 25)	72.0% (18)		52.0% (13)	
Capsular formation		0.028*		NS
Not available (n = 8)				
Yes (n = 59)	69.5% (41)		57.6% (34)	
No (n = 42)	88.1% (37)		66.7% (28)	
Micro-vascular thrombi		0.001*		0.019*
Yes (n = 48)	91.7% (44)		72.9% (35)	
No (n = 61)	65.6% (40)		50.8% (31)	
Liver cirrhosis		NS		NS
Not available (n = 3)				
Yes (n = 55)	76.4% (42)		58.2% (32)	
No (n = 51)	78.4% (40)		64.7% (33)	
Viral hepatitis		NS		NS
Not available (n = 7)				
Hepatitis B (n = 54)	79.6% (43)		61.1% (33)	
Hepatitis C (n = 30)	70.0% (21)		56.7% (17)	
Both (n = 15)	80.0% (12)		60.0% (9)	
None (n = 3)	66.7% (2)		66.7% (2)	
Alpha-fetoprotein level		NS		0.009*
Not available (n = 12)				
≧ 80 ng/ml (n = 35)	80.0% (28)		77.1% (27)	
< 80 ng/ml (n = 62)	74.2% (46)		50.0% (31)	
Subsequent extrahepatic metastasis		NS		NS
Yes (n = 29)	82.8%(24)		65.5% (19)	
No (n = 80)	75.0%(60)		58.8% (47)	

BCLC, Barcelona-Clinic Liver Cancer; NS, not significant; Q-score, Quick-score.

*p < 0.05; ^†^0.05 < *p* < 0.10.

### 14-3-3σ upregulates HSF-1 and HSP70 expression in HCC

To investigate the role of 14-3-3σ on HCC tumor progression, we examined the expression level of 14-3-3σ in HCC cell lines including Huh-7, HepG2, Hep3B, SK-Hep1 and PLC-5. Although 14-3-3σ was detectable in all tested cell lines, the expression of 14-3-3σ in Huh-7 was barely detected but there was strong expression in SK-Hep1, PLC-5 and HepG2 cells (Figure 1B). In addition, we examined 14-3-3σ levels in non-HCC cells, including human umbilical vein endothelial cells (HUVECs) and 293 cells, and found the expression of 14-3-3σ was undetectable in these cells (Additional file 1: Figure S1). We next established a stable HCC cell line with 14-3-3σ overexpression in Huh-7 cells. Huh-7 cells were transfected with p3XFlag-CMV (control) and p3XFlag-14-3-3σ (14-3-3σ) vectors and selected with G418 for 4 weeks. Single colonies were picked and expression of 14-3-3σ was confirmed by Western blot analysis of Flag and 14-3-3σ (Additional file 1: Figure S2A and B). Clone 2 of 14-3-3σ and clone 1 of the control were used for the following experiments. To examine whether 14-3-3σ regulates HCC growth, the cell proliferation rate was determined by an MTT assay. 14-3-3σ overexpression has no significant effect on cell proliferation in comparison to the control cells (Figure 1C).

**Figure 2:**
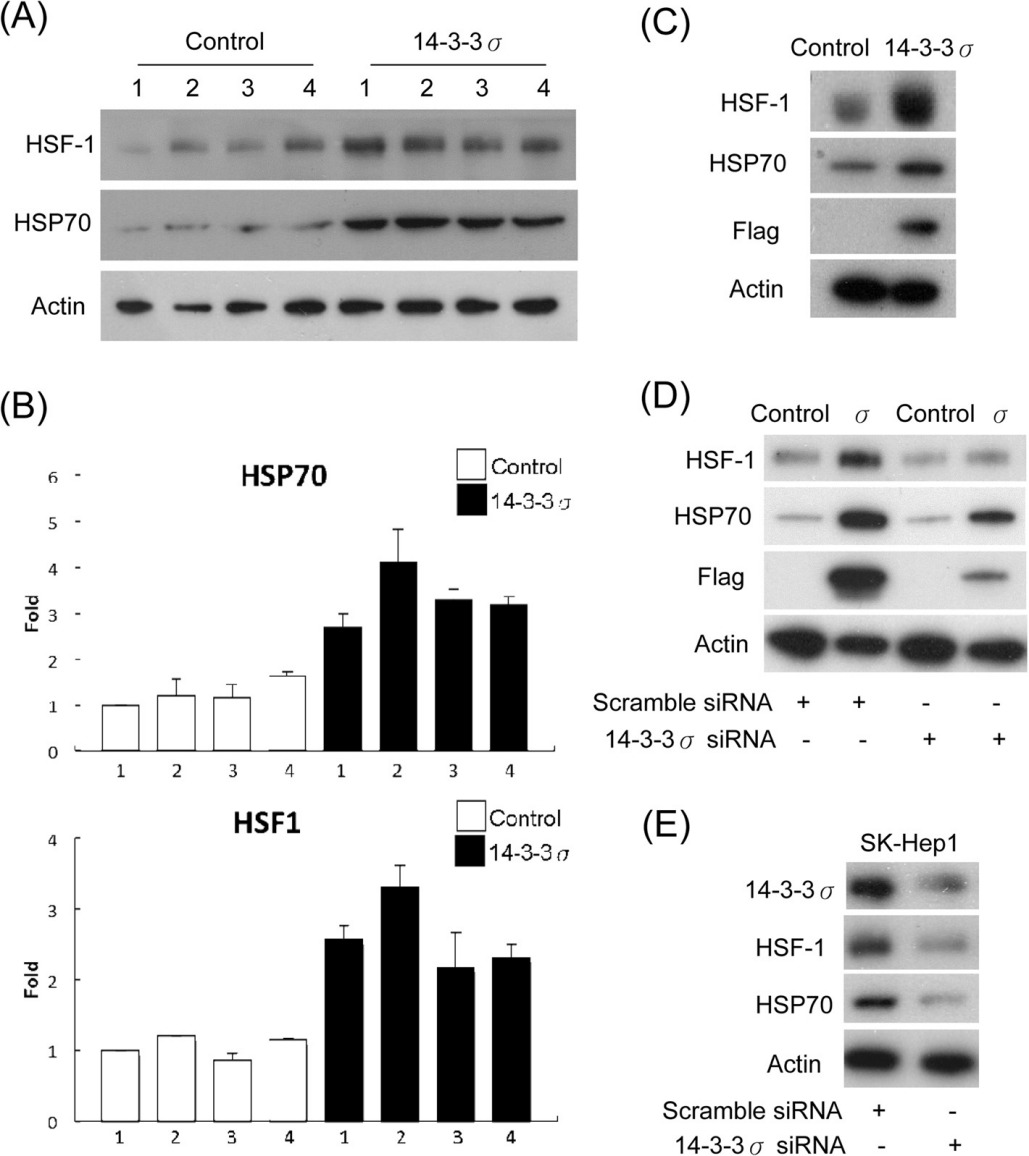
**14-3-3σ induces HSF-1 and HSP70 expression. (A)** Increased expression of HSF-1 and HSP70 induced by 14-3-3σ stable overexpression was determined by Western blotting and **(B)** real-time PCR analysis. Lanes 1–4 indicate as 4 different stable clones selected from the single colonies. Scale bars: mean ± SD. **(C)** Transient transfection of 14-3-3σ overexpression induced HSF-1 and HSP70 expression. **(D)** Knockdown of 14-3-3σ with siRNA suppressed HSF-1 and HSP70 expression in 14-3-3σ stable cells and **(E)** SK-Hep1 cells. Expression of HSF-1, HSP70, 14-3-3σ and Flag was determined by Western blot analysis. Actin was used as a loading control.

To explore the downstream factors of 14-3-3σ which are involved in modulating HCC tumor progression, we performed a gene expression profile analysis of 14-3-3σ overexpresion (Additional file 1: Table S3 and Table S4). Among the genes altered by 14-3-3σ overexpression, we identified HSP70 as one of the potential targets. Earlier studies have suggested that HSP70 serves as a potential biomarker for early detection of HCC [18-23]. To validate whether HSP70 expression is induced by 14-3-3σ, we determined HSP70 and its transcriptional activator, HSF-1α levels by Western blot analysis. Stable cells with increased 14-3-3σ expression significantly induced HSF-1α as well as HSP70 expression levels (Figure 2A). Induction of HSF-1α/HSP70 was further validated on the RNA level by quantitative real-time PCR analysis (Figure 2B). We next performed transient transfection experiments of 14-3-3σ overexpression in HCC cells. Transient overexpression of 14-3-3σ significantly induced HSF-1α/HSP70 expression in Huh-7 cells (Figure 2C). Furthermore, 14-3-3σ-induced HSF-1α and HSP70 expressions were attenuated by siRNA knockdown of 14-3-3σ (Figure 2D) and HSF-1α (Additional file 1: Figure S3). In addition, transfection of 14-3-3σ siRNA suppressed HSF-1α and HSP70 expressions in SK-Hep1 cells (Figure 2E). These results indicate that HSF-1α/HSP70 expressions are induced by 14-3-3σ in HCC.

### Association of 14-3-3σ expression with HSF-1α and HSP70 in HCC tissues

**Figure 3:**
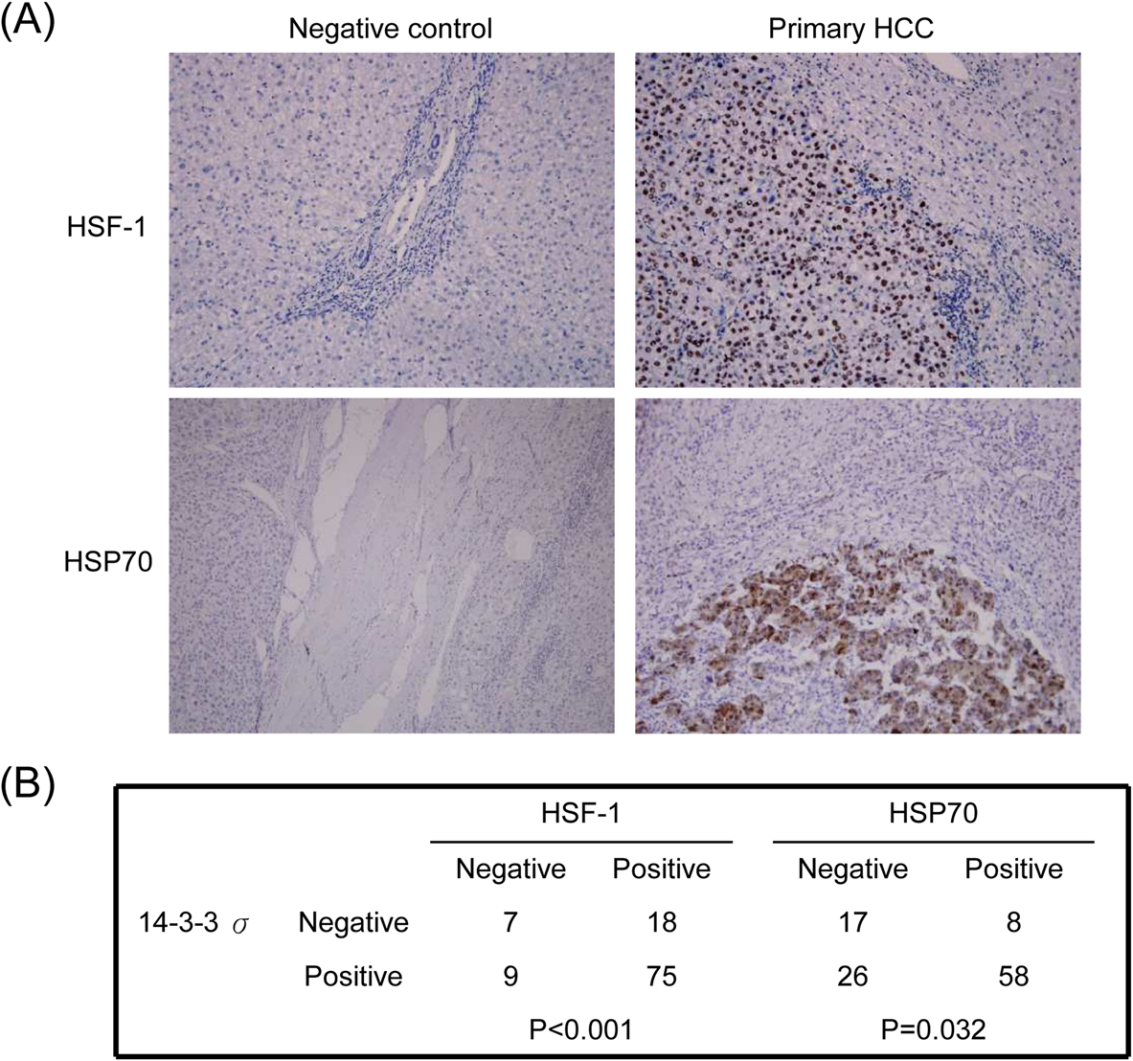
**Correlation of 14-3-3σ expression with HSF-1 and HSP70 in HCC tumors. (A)** Representative expression of HSF-1 and HSP70 in primary HCC tumors as well as negative control was examined by immunohistochemical analysis. Original magnification, ×200. **(B)** 14-3-3σ significantly correlates with HSF-1 and HSP70 expression in primary HCC tumors as analyzed by the Chi-square test.

To further show that 14-3-3σ induces HSF-1α/HSP70 in HCC, we examined and compared the expression of HSF-1α/HSP70 with 14-3-3σ by immunohistochemical staining in clinical HCC specimens. Both HSF-1α and HSP70 expression were correlatively increased in HCC tumors (Figure 3A). The expression of HSF-1α and HSP70 stained positively in 93 of 109 (85.3%) and 66 of 109 (60.6%) of primary HCC tumors, respectively. Furthermore, expression of 14-3-3σ positive tumors was significantly correlated with HSF-1α (*P* < 0.001) and HSP70 (*P* = 0.032) (Figure 3B). We next analyzed the correlation of HSP70 expression with clinicopathological characteristics and found that positive HSP70 expression was significantly associated with micro-vascular thrombi (*P* = 0.019) and alpha–fetoprotein levels (*P* = 0.009) (Table 1).

### 14-3-3σ/HSP70 enhanced HCC cell migration

**Figure 4:**
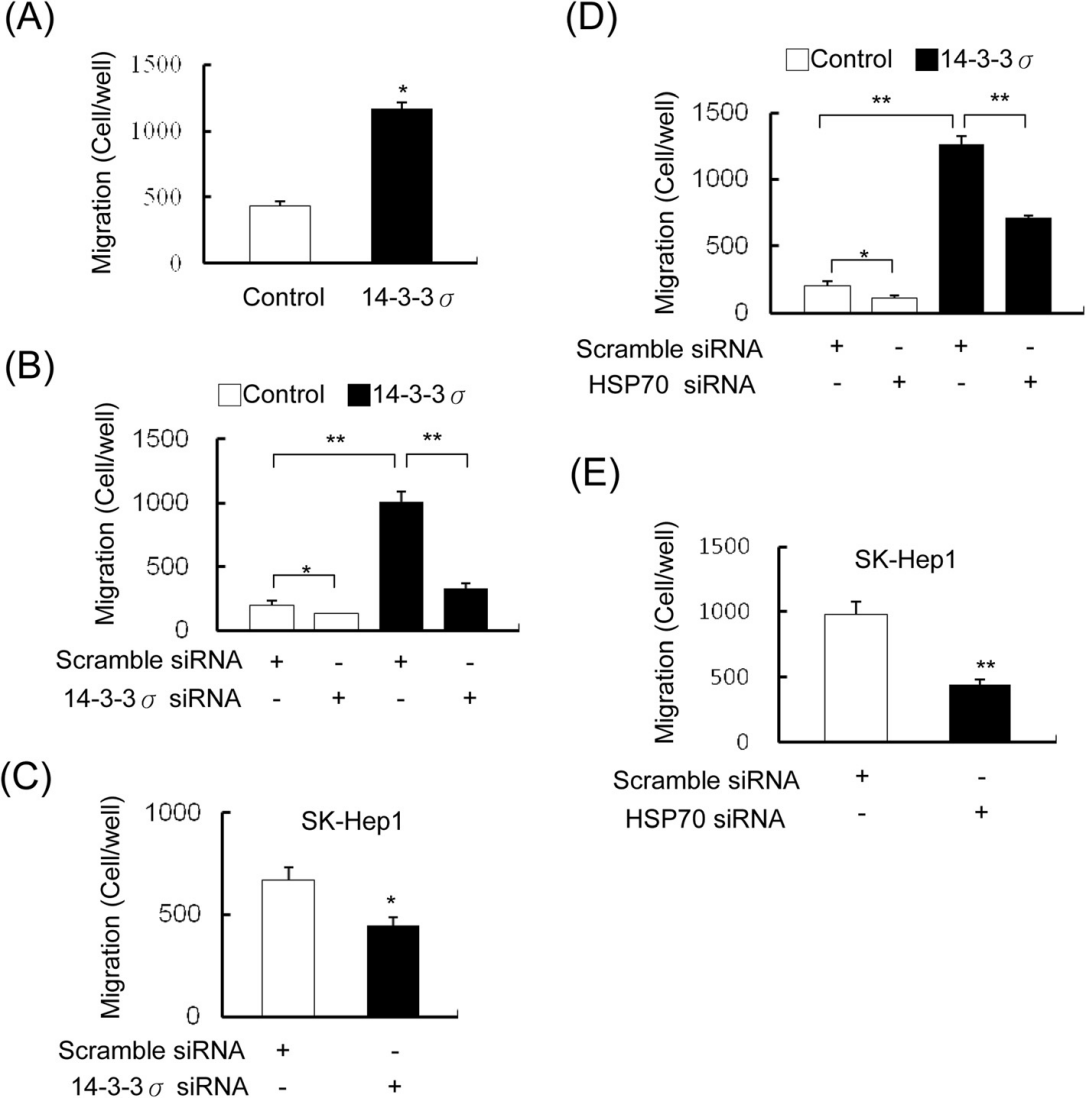
**14-3-3σ induces cell migration via an increase of HSP70 expression. (A)** Migratory ability of control and 14-3-3σ overexpressed Huh-7 cells was determined by a Boyden chamber assay. **(B)** Knockdown of 14-3-3σ with siRNA suppressed cell migration in 14-3-3σ stably overexpressed Huh-7 cells and **(C)** SK-Hep1 cells. **(D)** Knockdown of HSP70 with siRNA attenuated 14-3-3σ-induced cell migration in stable cells and **(E)** SK-Hep1 cells. Scale bars: mean ± SD. *P < 0.05, **P < 0.01.

While comparing the expression of 14-3-3σ and HSP70 with clinicopathological parameters, the most significant factor is the appearance of micro-vascular thrombi which positivity correlates with both 14-3-3σ and HSP70 (Table 1). Increased micro-vascular thrombi imply enhanced tumor migration/invasion. We therefore wanted to determine whether HSP70 expression influences 14-3-3σ-induced cell migration/invasion. We first investigated whether 14-3-3σ overexpression affects cell migration/invasion. 14-3-3σ overexpression significantly facilitates cell migration (Figure 4A), although an unexpected effect of suppressing invasion was found (Additional file 1: Figure S4). Moreover, the 14-3-3σ induced migratory ability was significantly abrogated by siRNA knockdown in both of the 14-3-3σ stable cells (Figure 4B) and SK-Hep1 cells (Figure 4C). These results indicate that increased 14-3-3σ expression results in promoting HCC cell migration. To further investigate whether HSP70 is involved in 14-3-3σ-induced cell migration, SK-Hep1 cells overexpressing 14-3-3σ were transfected with HSP70 siRNAs. Knockdown of HSP70 significantly impaired 14-3-3σ-induced cell migration in stable (Figure 4D) and in SK-Hep1 cells (Figure 4E). These results indicate that HSP70 is an important downstream regulator of 14-3-3σ which in turn regulates HCC cell migration and tumor progression.

### 14-3-3σ induces HSF-1α expression via a GSK-3β/β-catenin dependent mechanism

**Figure 5:**
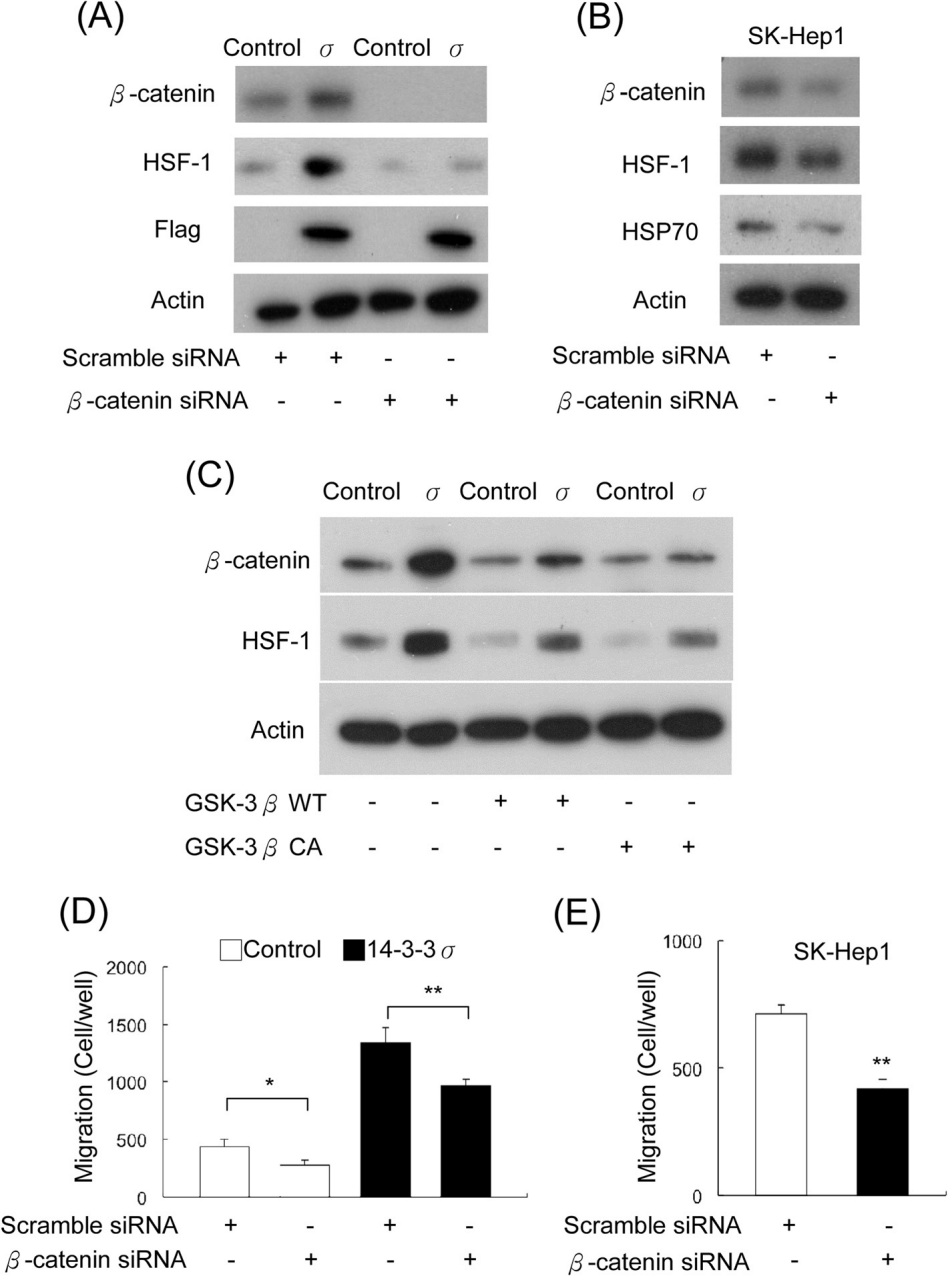
**14-3-3σ induces HSF-1 expression via regulating β-catenin. (A)** 14-3-3σ-induced HSF-1 expression was abolished by knockdown of β-catenin in stable cells and **(B)** SK-Hep1 cells. **(C)** 14-3-3σ-induced HSF-1 expression was attenuated by overexpression with wild type (WT) and constitutively activated (CA) mutant of GSK-3β. Expression of β-catenin, HSF-1, HSP70 and Flag was determined by Western blot analysis. Actin was used as a loading control. **(D)** Migratory ability was determined by a Boyden chamber assay. Knockdown of β-catenin with siRNA suppressed cell migration in 14-3-3σ stably overexpressed cells and **(E)** SK-Hep1 cells. Scale bars: mean ± SD. *P < 0.05, **P < 0.01.

HSP70 is transcriptionally upregulated by HSF-1α activation. The molecular pathway controlling HSF-1α expression has never been well characterized. Earlier studies indicate that GSK-3β modulates HSF-1α activity [29-31] and the stability of β-catenin is known to be the major downstream event regulated by GSK-3β. We thus postulated that GSK-3β/β-catenin may be involved in regulating 14-3-3σ-induced HSF-1α/HSP70 expression. To prove this hypothesis, 14-3-3σ overexpressing and control cells were transfected with β-catenin or scramble siRNA and the expression of HSF-1α was determined by Western blot analysis. Knockdown of β-catenin significantly abrogates 14-3-3σ-induced HSF-1α expression (Figure 5A). The effects of HSF-1α and HSP70 suppressive expressions by β-catenin knockdown were also confirmed in SK-Hep1 cells (Figure 5B). In addition, we found that 14-3-3σ overexpression increases β-catenin expression levels (Figure 5A and C). Transient activation of GSK-3β by transfection of wild type (GSK-3β WT) or constitutively activated mutant (GSK-3β CA) significantly attenuates 14-3-3σ–induced β-catenin and HSF-1α expression (Figure 5C). We next examined whether β-catenin is involved in regulating cell migration of HCC. The 14-3-3σ/control cells and SK-Hep1 cells were transfected with siRNA of β-catenin and the migration ability was determined by a trans-well assay. Knockdown of β-catenin significantly reduced cell migration of 14-3-3σ/control cells (Figure 5D) and SK-Hep1 cells (Figure 5E). These results suggest that 14-3-3σ induced HSF-1α/HSP70 expression and cell migration is mediated by the GSK-3β/β-catenin signaling pathway.

## Discussion

Our results show that 14-3-3σ plays major roles in HCC. We initially showed that 14-3-3σ is overexpressed in HCC tumors and that HSP70, one of the downstream factors of 14-3-3σ, is upregulated by 14-3-3σ and this action is mediated by the GSK-3β/β-catenin/HSF-1α signaling pathway. Additionally, 14-3-3σ modulates HSP70 to promote cell migration in HCC. These findings suggest that 14-3-3σ together with β-catenin/HSF-1α and HSP70 enhance HCC tumor progression. 14-3-3σ was shown to both enhance and restrict malignant tumor progression in different tumor types pointing to a cell type specific effect of 14-3-3σ. For instance, 14-3-3σ is frequently DNA hypermethylation and subsequently silenced in lung cancers. However, variations of 14-3-3σ expression levels were found in heterogeneous lung cancers [44]. Recent studies have demonstrated that a highly methylated *SFN* promoter is found in normal lung tissue and in adenocarcinomas [13], however, 14-3-3σ is overexpressed in early invasive adenocarcinoma [13,15]. These studies indicate that 14-3-3σ expression in tumors is modulated by a complicated regulatory mechanism. In addition, analysis of 14-3-3σ and HSP70 expressions with clinicopathological parameters reveal that both 14-3-3σ and HSP70 are correlated with micro-vascular thrombi in HCC patients. We have shown that 14-3-3σ overexpression induces HCC cell migration (Figure 4A) but unexpectedly reduces cell invasion (Additional file 1: Figure S4). These results reveal that the 14-3-3σ promotion of HCC cell invasion and tumor metastasis are complicated processes and other essential synergistic regulators are probably involved. By simply elevating 14-3-3σ expression without activating other synergistic factors may result in a reciprocal phenomenon. This may also explain the duplicitous role of 14-3-3σ reported in earlier studies [16,17]. In addition, since extracellularly secreted 14-3-3σ has been shown to affect muscle remodeling in keratinocyte associated fibroblasts [44], we postulate that the surrounding stromal cells associated with tumors play important roles in 14-3-3σ-promoting HCC tumor progression. 14-3-3σ might synergize with other signals from its micro-environment milieu and tumor-stromal interactions may therefore be critical for tumor progression. Further investigation needs to be done to ascertain if 14-3-3σ combines with additional factors and if 14-3-3σ’s expression in tumor heterogeneity is found in distinct stages of HCC.

We found that either the moderate (clone 1 and 2) or extremely higher (clone 3 and 4) expression of 14-3-3σ (Additional file 1: Figure S2B) upregulate the expression of HSF-1α and HSP70 (Figure 2A and B). These results suggest that the appropriately increased 14-3-3σ expression may be sufficient to regulate HCC tumor progression and an excess of 14-3-3σ have no further effect on HSF-1α and HSP70 expression (Figure 2A and B).

In this work, we show for the first time that HSF-1α/HSP70 is regulated by β-catenin in HCC. β-catenin is an important transcriptional regulator in promoting cell proliferation. Our recently published data indicates that 14-3-3σ promotes mouse embryonic stem cell proliferation via enhancing β-catenin stability [45], suggesting that 14-3-3σ may modulate the β-catenin signal pathway to promote cell proliferation. In addition, earlier studies have indicated that β-catenin signaling regulates GS expression and is involved in glutamine metabolism [46,47]. Since β-catenin is known to be a pivotal factor in promoting HCC development [34-37], both HSF-1α/HSP70 and GS are potential downstream targets of β-catenin. Whether the potential TCF/LEF binding motifs found on the promoters of HSF-1 and GS are regulated by β-catenin needs further investigation.

## Conclusion

Our results link 14-3-3σ with β-catenin/HSF-1α/HSP70 and these regulators work as a network in promoting HCC development. We also provide evidence at the cellular and clinical tissue levels to suggest that 14-3-3σ is the upstream modulator of β-catenin and HSF-1α/HSP70 in HCC. 14-3-3σ combined with other potential markers including HSP70 might be used as potential prognostic biomarkers for HCC.

## Competing interests

The authors declare that they have no competing interests.

## Authors’ contributions

CCL, YJJ, BSK, SML, SCC, YML, TAL, TCC conducted experiments; JYL, YJJ, BSK, CCL, LYS participated in the design of this experiments; JYL, YJJ, BSK, YMW, JW, SKS, LYS analyzed the data; JYL wrote the manuscript; All authors read and approved the final manuscript.

## Pre-publication history

The pre-publication history for this paper can be accessed here:

http://www.biomedcentral.com/1471-2407/14/425/prepub

## Supplementary Material

Additional file 1 **Table S1.** Oligonucleotide sequences for Q-PCR. **Table S2**. Oligonucleotide sequences of small interfering RNAs. **Table S3**. Gene expression (>4 fold) induced by 14-3-3σ overexpression in Huh-7 stable cells was analyzed by microarray analysis. The total RNA samples were extracted from control and 14-3-3σ overexpressed cells using Qiagen RNeasy Mini Kit (Qiagen, Valencia, CA) and the microarray analysis was processed according to the manufacturers’ instructions of Affymetrix Inc. (Santa Clara, CA). **Table S4**. Gene expression (<4 fold) suppressed by 14-3-3σ overexpression was analyzed by microarray analysis. **Figure S1.** Expression of 14-3-3σ in non-HCC (HUVECs and 293) and HCC (Huh-7, HepG2 and SK-Hep1) cells was determined by Western blotting analysis. Actin was used as loading control. **Figure S2.** Establishment of 14-3-3σ stable cell lines. Huh-7 cells were transfected with p3XFlag-CMV (control) and Flag-tagged 14-3-3σ overexpression vectors, followed by selection with G418 for 4 Weeks. Expression of 14-3-3σ in stable cells was confirmed by Western blot analysis of (A) Flag and (B) 14-3-3σ (4 clones for each of control and 14-3-3σ). Actin was used as loading control. **Figure S3.** 14-3-3σ-induced HSP70 expression was attenuated by knockdown of HSF-1 with siRNA. Control and 14-3-3σ stable cells were transfected with scramble or HSF-1 siRNA. Expression of HSF-1 and HSP70 was determined by Western blotting analysis. Actin was used as loading control. **Figure S4.** 14-3-3σ reduces cell invasion. Efficacy of cell invasion was examined by two-chamber analysis. Click here for file
