# Supplementary material for: 14-3-3σ induces heat shock protein 70 expression in hepatocellular carcinoma
Source: BMC Cancer. 2014 Jun 12;14:425. doi: 10.1186/1471-2407-14-425 (PMC4061114; doi:10.1186/1471-2407-14-425)
Supplement: Additional file 1 — Table S1. Oligonucleotide sequences for Q-PCR. Table S2. Oligonucleotide sequences of small interfering RNAs. Table S3. Gene expression (>4 fold) induced by 14-3-3σ overexpression in Huh-7 stable cells was analyzed by microarray analysis. The total RNA samples were extracted from control and 14-3-3σ overexpressed cells using Qiagen RNeasy Mini Kit (Qiagen, Valencia, CA) and the microarray analysis was processed according to the manufacturers’ instructions of Affymetrix Inc. (Santa Clara, CA). Table S4. Gene expression (<4 fold) suppressed by 14-3-3σ overexpression was analyzed by microarray analysis. Figure S1. Expression of 14-3-3σ in non-HCC (HUVECs and 293) and HCC (Huh-7, HepG2 and SK-Hep1) cells was determined by Western blotting analysis. Actin was used as loading control. Figure S2. Establishment of 14-3-3σ stable cell lines. Huh-7 cells were transfected with p3XFlag-CMV (control) and Flag-tagged 14-3-3σ overexpression vectors, followed by selection with G418 for 4 Weeks. Expression of 14-3-3σ in stable cells was confirmed by Western blot analysis of (A) Flag and (B) 14-3-3σ (4 clones for each of control and 14-3-3σ). Actin was used as loading control. Figure S3. 14-3-3σ-induced HSP70 expression was attenuated by knockdown of HSF-1 with siRNA. Control and 14-3-3σ stable cells were transfected with scramble or HSF-1 siRNA. Expression of HSF-1 and HSP70 was determined by Western blotting analysis. Actin was used as loading control. Figure S4. 14-3-3σ reduces cell invasion. Efficacy of cell invasion was examined by two-chamber analysis. [file 1471-2407-14-425-S1.doc]

**Additional file 1**

**Table S1**. Oligonucleotide sequences for Q-PCR

| **Gene** | Primers Sequences |
| --- | --- |
| HSF1 | Fw 5’- ACCCATGCTTCCTGCGTGGC -3’  Rv 5’- TGCTTCTGCCGAAGGCTGGC -3’ |
| HSP70 | Fw 5’- TTTGAGGGCATCGACTTCTACA -3’  Rv 5’- CCAGGACCAGGTCGTGAATC -3’ |

**Table S2**. Oligonucleotide sequences of small interfering RNAs

| **Accession no.** | | **Gene name** | **siRNA primer sequences** | **Concentration**  **(nm)** |  |
| --- | --- | --- | --- | --- | --- |
| NM_006142.3 | | SFN (14-3-3σ) | Fw 5’- UCUCAGUAGCCUAUAAGAACGUGGU -3’  Rv 5’- AGAGUCAUCGGAUAUUCUUGCACCA -3’ | 20 |  |
| [NM_002154.3](http://www.ncbi.nlm.nih.gov/nuccore/301336132) | | HSPA4 (HSP70) | sequence 1  Fw 5’- GCAUCCCCAAGGUGCAGAATT -3’  Rv 3’- UUCUGCACCUUGGGGAUGCTT -5’  sequence 2  Fw 5’- CCUGUGUUUGCAAUGUUATT -3’  Rv 3’- UCAACAUUGCAAACACAGGTT -5’ sequence 3  Fw 5’- CUGUGUUUGCAAUGUUGAATT -3’  Rv 3’- UUCAACAUUGCAAACACAGTT -5’ sequence 4  Fw 5’- GUUGGUUACUUCAAAGUAATT -3’  Rv 3’- UUACUUUGAAGUAACCAACTT -5’ | 20 |  |
| NM_005526.2 | | HSF1  (HSF1) | sequence 1  Fw 5’- CCCAUCAUCUCCGACAUCATT -3’  Rv 3’-UGAUGUCGGAGAUGAUGGGTT -5’ | 20 |  |
|  | |  | sequence 2  Fw 5’-GUGACCACUUGGAUGCUAUTT-3’  Rv 3’-AUAGCAUCCAAGUGGUCACTT-5’ |  |  |
|  | |  | sequence 3  Fw 5’-CGUGUCCUGUGGUUUGGUUTT-3’  Rv 3’-AACCAAACCACAGGACACGTT-5’ |  |  |
| NM_001012271.1 | | CTNNB1  (β-catenin) | Fw 5’- AGCUGAUAUUGAUGGACAGTT -3’  Rv 3’- CUGUCCAUCAAUAUCAGCUTT-5’ |  |  |
|  |  | | | | |

**Table S3**. Gene expression (>4 fold) induced by 14-3-3σ overexpression in Huh-7 stable cells was analyzed by microarray analysis. The total RNA samples were extracted from control and 14-3-3σ overexpressed cells using Qiagen RNeasy Mini Kit (Qiagen, Valencia, CA) and the microarray analysis was processed according to the manufacturers’ instructions of Affymetrix Inc. (Santa Clara, CA).


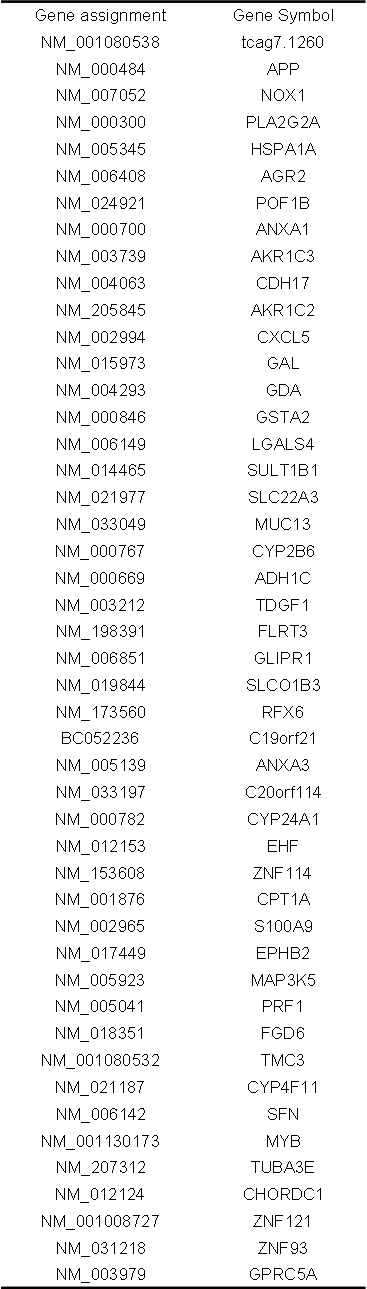


**Table S4**. Gene expression (<4 fold) suppressed by 14-3-3σ overexpression was analyzed by microarray analysis.


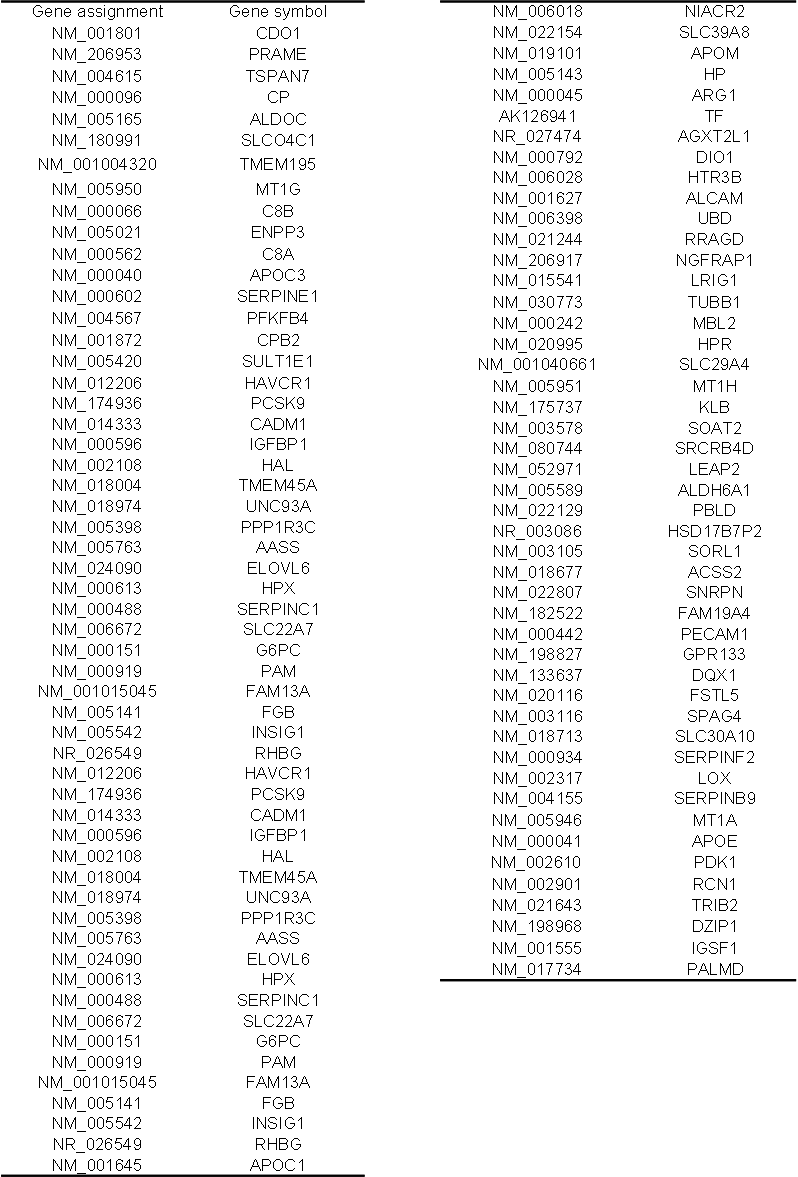


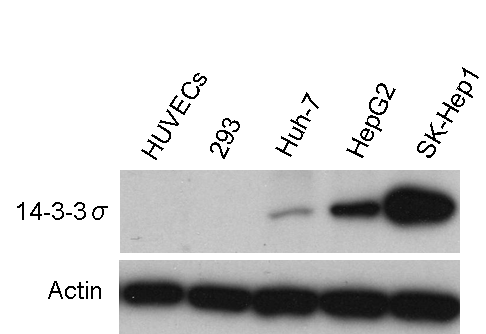


**Figure S1.** Expression of 14-3-3σ in non-HCC (HUVECs and 293) and HCC (Huh-7, HepG2 and SK-Hep1) cells was determined by Western blotting analysis. Actin was used as loading control.


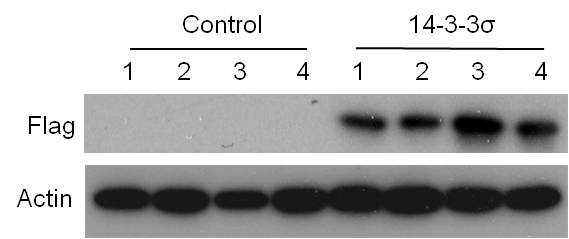
(A)

(B)

**
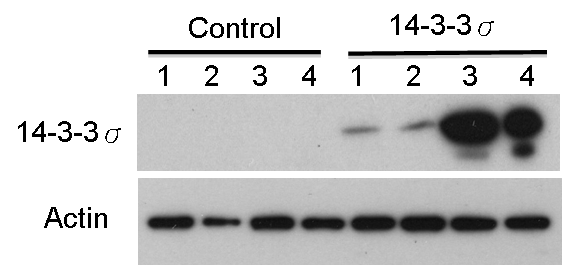
**

**Figure S2.** Establishment of 14-3-3σ stable cell lines. Huh-7 cells were transfected with p3XFlag-CMV (control) and Flag-tagged 14-3-3σ overexpression vectors, followed by selection with G418 for 4 Weeks. Expression of 14-3-3σ in stable cells was confirmed by Western blot analysis of (A) Flag and (B) 14-3-3σ (4 clones for each of control and 14-3-3σ). Actin was used as loading control.

**
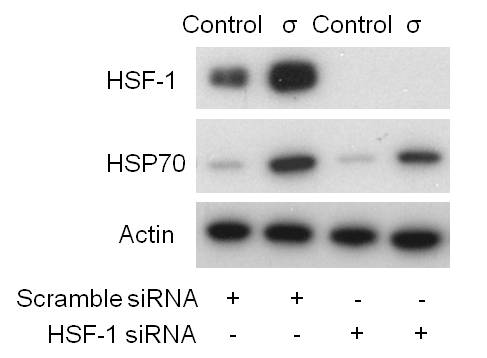
**

**Figure S3.** 14-3-3σ-induced HSP70 expression was attenuated by knockdown of HSF-1 with siRNA. Control and 14-3-3σ stable cells were transfected with scramble or HSF-1 siRNA. Expression of HSF-1 and HSP70 was determined by Western blotting analysis. Actin was used as loading control.

**
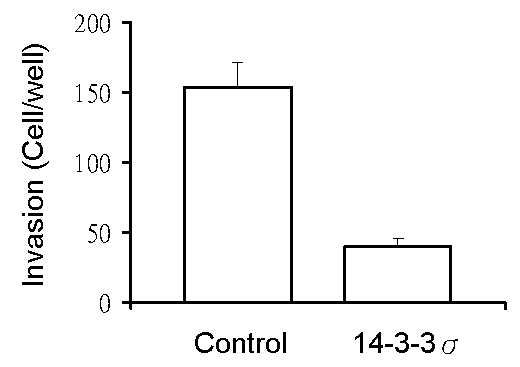
**

**Figure S4.** 14-3-3σ reduces cell invasion. Efficacy of cell invasion was examined by two-chamber analysis.
